# Near-infrared-traceable DNA nano-hydrolase: specific eradication of telomeric G-overhang *in vivo*

**DOI:** 10.1093/nar/gkaa693

**Published:** 2020-08-27

**Authors:** Yuhuan Sun, Chuanqi Zhao, Tingting Cui, Hongshuang Qin, Jingsheng Niu, Jinsong Ren, Xiaogang Qu

**Affiliations:** Laboratory of Chemical Biology and State Key Laboratory of Rare Earth Resource Utilization, Changchun Institute of Applied Chemistry, Chinese Academy of Sciences, Changchun, Jilin 130022, China; School of Applied Chemistry and Engineering, University of Science and Technology of China, Hefei, Anhui 230026, China; Laboratory of Chemical Biology and State Key Laboratory of Rare Earth Resource Utilization, Changchun Institute of Applied Chemistry, Chinese Academy of Sciences, Changchun, Jilin 130022, China; Laboratory of Chemical Biology and State Key Laboratory of Rare Earth Resource Utilization, Changchun Institute of Applied Chemistry, Chinese Academy of Sciences, Changchun, Jilin 130022, China; School of Applied Chemistry and Engineering, University of Science and Technology of China, Hefei, Anhui 230026, China; Laboratory of Chemical Biology and State Key Laboratory of Rare Earth Resource Utilization, Changchun Institute of Applied Chemistry, Chinese Academy of Sciences, Changchun, Jilin 130022, China; Laboratory of Chemical Biology and State Key Laboratory of Rare Earth Resource Utilization, Changchun Institute of Applied Chemistry, Chinese Academy of Sciences, Changchun, Jilin 130022, China; School of Applied Chemistry and Engineering, University of Science and Technology of China, Hefei, Anhui 230026, China; Laboratory of Chemical Biology and State Key Laboratory of Rare Earth Resource Utilization, Changchun Institute of Applied Chemistry, Chinese Academy of Sciences, Changchun, Jilin 130022, China; School of Applied Chemistry and Engineering, University of Science and Technology of China, Hefei, Anhui 230026, China; Laboratory of Chemical Biology and State Key Laboratory of Rare Earth Resource Utilization, Changchun Institute of Applied Chemistry, Chinese Academy of Sciences, Changchun, Jilin 130022, China; School of Applied Chemistry and Engineering, University of Science and Technology of China, Hefei, Anhui 230026, China

## Abstract

Telomeric DNA, whose length homeostasis is closely correlated with immortality of cancer cells, is regarded as a molecular clock for cellular lifespan. Regarding the capacity in forming G-quadruplex, G-rich 3′-overhang (G-overhang) has been considered as an attractive anticancer target. However, it is still challenging to precisely target telomeric G-overhang with current ligands because of the polymorphism of G-quadruplexes in cells. Herein, we construct a telomeric G-overhang-specific near-infrared-traceable DNA nano-hydrolase, which is composed of four parts: (i) dexamethasone for targeting cell nuclei; (ii) complementary DNA for hybridizing with G-overhang; (iii) multinuclear Ce(IV) complexes for hydrolyzing G-overhang; and (iv) upconversion nanoparticles for real-time tracking. The multivalent targeted DNA nano-hydrolase can be traced to precisely digest telomeric G-overhang, which contributes to telomeric DNA shortening and thereby causes cell aging and apoptosis. The anticancer treatment is further proved by *in vivo* studies. In this way, this design provides a telomeric G-overhang-specific eradication strategy based on a non-G-quadruplex targeting manner.

## INTRODUCTION

Telomere caps chromosome ends and protects them from end-to-end fusion, recombination and degradation (1). It is composed of telomeric DNA and DNA-binding proteins. In normal cells, telomeric DNA is shortened by each cell division until a critical length is achieved, and then this triggers the cell senescence and eventually cell death. Telomeric DNA is aging and disease related, and regarded as a molecular clock for cellular lifespan. In contrast, telomeric DNA length in tumor cells is stably maintained by either the action of telomerase or the alternative lengthening of telomere mechanism (2). Immortality of cancer cells is closely correlated with their telomere length homeostasis, which, however, would be disrupted by accelerating telomere shortening (3). Currently, the attrition of telomere DNA has been developed into a potential strategy for cancer treatment (4–7).

Telomeric DNA is terminated by a protruding G-rich 3′-overhang (G-overhang) ranging from 100 to 300 nucleotides (8). It plays pivotal role in regulating telomeric DNA length and guarding the structural integrity of chromosome and genomic stability by formation of shelterin and T-loop structure with the duplex region. G-overhang is an attractive target for anticancer treatment. Under physiological conditions, G-overhang has the ability to fold into polymorphic G-quadruplex structures by stacking of G-tetrad layers (9). G-quadruplex formation can inhibit telomerase activity, and thus telomeric G-quadruplex has been an attractive target for the development of anticancer drugs (10,11). Currently, the design of chemical ligands especially stabilizing telomeric G-quadruplex is still the most prevalent strategy in this field (12–14). However, recent studies indicate that up to 716 310 DNA sequences in human genome have the propensity to form G-quadruplex structure (15). Therefore, it is still rather challenging for current G-quadruplex ligands to precisely target telomeric G-quadruplex among such a huge number of G-quadruplexes in genome (16). In this regard, except for developing novel telomeric G-quadruplex-specific ligands, alternative G-overhang-targeted strategy for telomeric DNA attrition is highly demanded.

**Figure 1.**
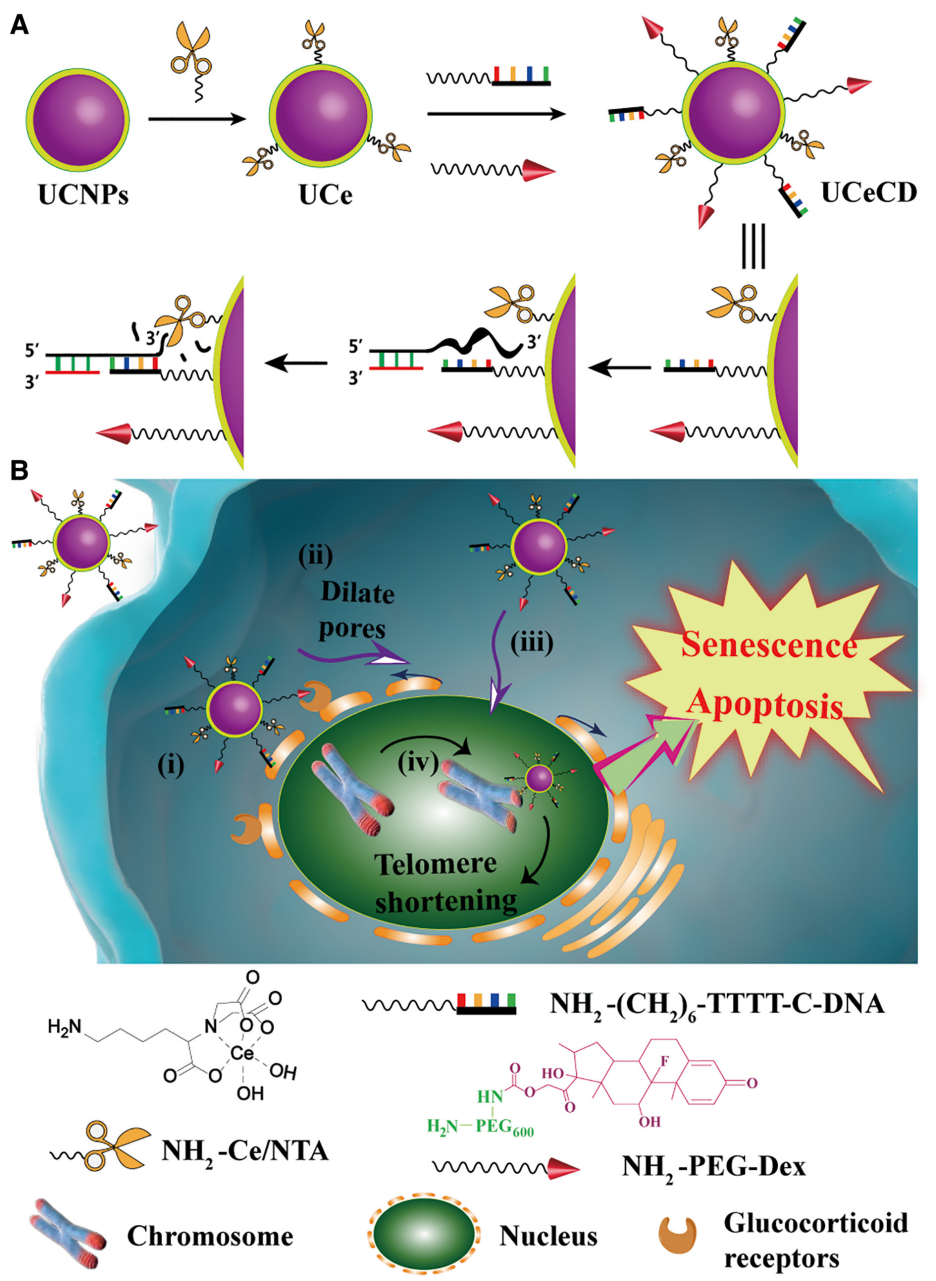
Schematic illustration of G-overhang-targeted DNA nano-hydrolase for anticancer application. (**A**) Preparation route of DNA nano-hydrolase (UCeCD) and schematic illustration of telomeric DNA hydrolysis based on UCeCD are given. (**B**) The path of action of UCeCD on mammalian cancer cells: (i) nuclear localization; (ii) dilation of nuclear pores; (iii) translocation from cytoplasm to nuclei; and (iv) targeting G-overhang. Then, telomere shortening is realized by the multinuclear cooperative hydrolysis effects. Finally, cell senescence and apoptosis occur, leading to a significant reduction in cell survival.

Herein, a multivalent telomeric G-overhang-specific near-infrared nano-nuclease mimic, named UCeCD, was constructed. As shown in Figure 1, UCeCD is composed of four parts: dexamethasone (Dex) for targeting cell nuclei; complementary DNA (C-DNA) for hybridizing with G-overhang; Ce(IV) nitrilotriacetic acid (NTA) complexes (Ce/NTA) for hydrolyzing G-overhang; and near-infrared upconversion nanoparticles (UCNPs) for real-time tracking. By controlling the linker length, the external Dex first targets glucocorticoid receptors, which are nuclear receptors expressed in various cancer cells (17–20), and, interestingly, Dex can dilate the nuclear pore to 60 nm to facilitate the entry of nanoparticles into the nucleus (21,22). Afterward, C-DNA on UCNPs specifically hybridizes with G-overhang. Then, by means of multivalent coordination, the near-infrared nano-nuclease mimic can trace and effectively hydrolyze G-overhang (23–25). Ultimately, the shortened G-overhang induces telomeric DNA shortening and damage, causing cell apoptosis and senescence.

## MATERIALS AND METHODS

### Materials

NH_4_F, octadecene, oleic acid and bis(4-nitrophenyl) phosphate sodium salt (BNPP) were purchased from Sigma-Aldrich. YbCl_3_·6H_2_O, YCl_3_·6H_2_O, TmCl_3_·6H_2_O, dopamine hydrochloride, NaOH, triethylamine, *N,N*′-disuccinimidyl carbonate, Dex, bromoacetic acid, Pd/C catalyst, *N*-ethylmorpholine and ammonium ceric nitrate (Ce(NH_4_)_2_(NO_3_)_6_) were obtained from Aladdin Reagent (Shanghai, China). Methanol, Triton X-100, ammonium hydroxide, hexanol and cyclohexane were provided by Beijing Chemicals (Beijing, China). *N*^ϵ^-Benzyloxycarbonyl-l-lysine was supplied by Xiya Reagent. The rabbit polyclonal antibody against γ-H_2_AX (Ser139) was obtained from Bioss (Beijing, China). The mouse monoclonal antibody against TRF1 was obtained from Santa Cruz Biotechnology (USA). DNA was synthesized by Shanghai Sangon Biological Engineering Technology & Services (Shanghai, China). The concentrations of DNA were determined by ultraviolet absorbance measurements (260 nm). All DNA sequences are listed in Supplementary Table S2. Chemicals were purchased from Sigma-Aldrich and used without further purification. All water used to prepare buffer solutions was obtained by using a Milli-Q water system.

### Catalytic activity measurement of nano-hydrolase toward the DNA model substrate (23)

BNPP was utilized as a DNA model substrate to study the catalytic activity of UCNPs@Ce (named UCe). First, the calibration curve of nitrophenolate in phosphate-buffered saline (PBS) buffer (pH 8.0, 10 mM) was determined by absorbance at 400 nm. Second, the hydrolysis rate of BNPP catalyzed by UCe in Tris buffer (pH 8.0) was measured by the increase in 400 nm absorption of nitrophenolate. The reaction solutions contained 0.1 mg/ml UCe and 0–0.6 mM BNPP. The kinetic data were collected using the initial slope method. From the slope and the concentration of BNPP, the pseudo-first-order rate constant *k*_obs_(BNPP) (s^−1^) was determined. All reactions were repeated at least three times.

### Cleavage of telomeric DNA

Cleavage reaction of telomeric DNA by artificial hydrolase was performed in Tris buffer (pH 7.4) at 37°C. At predetermined time intervals, the reaction mixtures were analyzed by denaturing 15% polyacrylamide gel electrophoresis. [DNA] = 10 μM; [UCNPs] = 0–120 μg/ml. Gels were run in TB buffer at room temperature and stained by stains-all dye. To further verify the sequence-targeting cleavage ability of UCeCD, FAM-labeled telomere DNA was utilized. In fluorescence measurements of supernatant of reaction mixture, the samples were excited at 470 nm, and emission spectra were collected from 480 nm to 600 nm.

### Statistical analysis

All experiments shown were performed at least three independent times. All data were expressed in this article as mean ± standard deviation. Sample sizes were statistically obtained by Nano Measurer software according to transmission electron microscopy (TEM) photos. The statistical analysis was performed by using Origin 8.0 software.

## RESULTS AND DISCUSSION

### Material characterization

**Figure 2.**
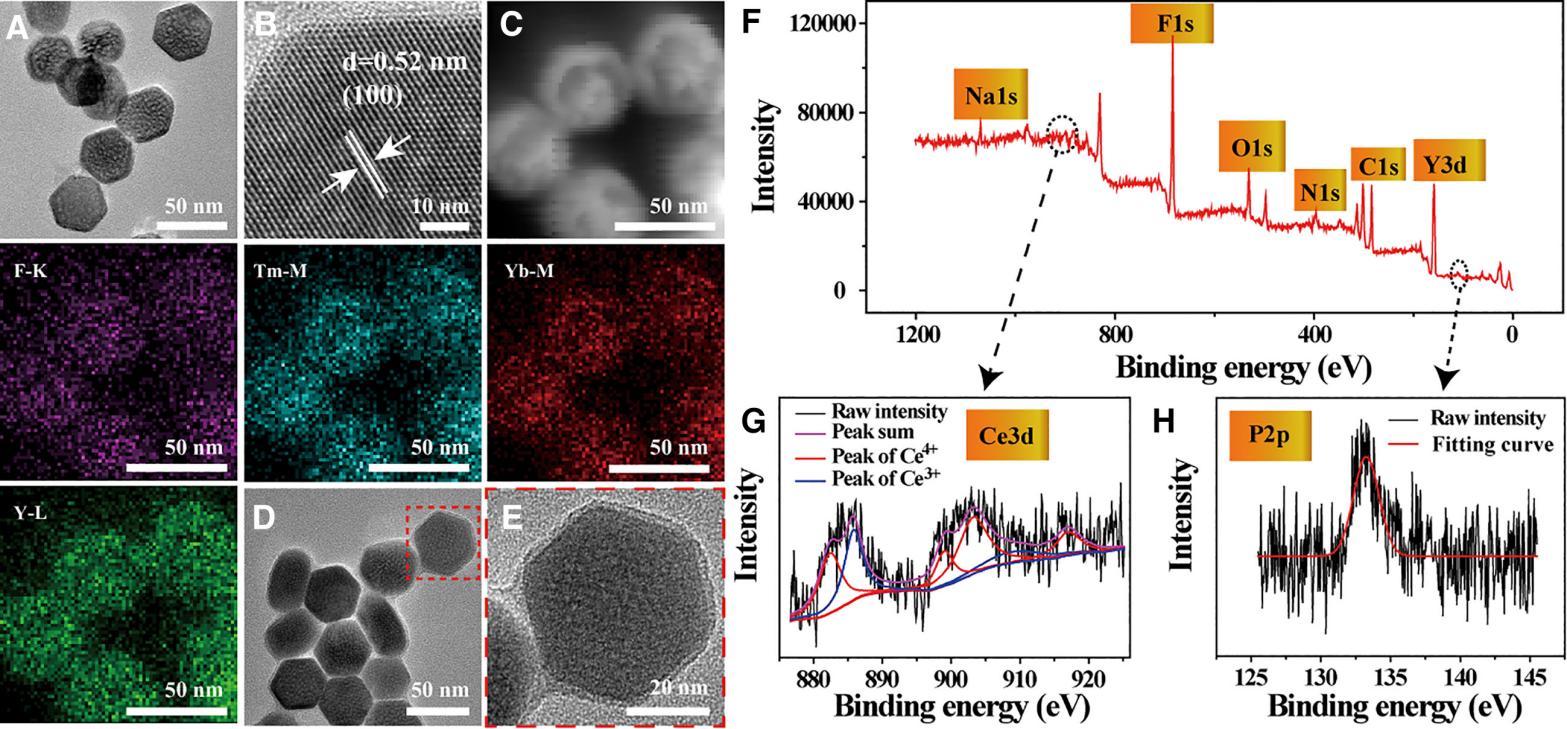
Material characterization. (**A**) TEM and (**B**) high-resolution TEM images of NaYF_4_:TmYb. (**C**) Dark-field TEM image of UCNPs and the corresponding TEM elemental mappings of F-K, Tm-M, Yb-M and Y-L edge signals. (**D**) TEM images of UCNPs@PDA and (**E**) the enlarged TEM images of red frame in (D). (**F**) X-ray photon spectroscopy (XPS) analysis of UCeCD. (**G**, **H**) XPS Ce 3d analysis and P2p analysis, respectively.

For construction of UCeCD, UCNPs as a core were synthesized through thermal decomposition (26,27). An average diameter of 33.2 nm was estimated in TEM images (Figure 2A and Supplementary Figure S1A and B). The typical *d*-spacing value of 0.52 nm was observed in accordance with the crystal structure of UCNPs (JCPDS = 28-1192; Figure 2B and Supplementary Figure S1C), proving a single hexagonal phase crystal (28). Meanwhile, TEM elemental mapping confirmed the composition of UCNPs (Figure 2C). Next, for further biocompatible modification, an ultrathin polydopamine (PDA) film (∼3 nm) was assembled on the surface of UCNPs (UCNPs@PDA) through a water-in-oil microemulsion method (Figure 2D and E, and Supplementary Figure S1D) (29). Importantly, the encapsulation of PDA had no obvious effect on the upconversion luminescence (UCL) of UCNPs (Supplementary Figure S1E and F), which was favorable for real-time tracking of the location of nanoparticles in cells.

For Ce/NTA, an NTA derivative ((1*S*)-*N*-(5-amino-1-carboxypentyl)iminodiacetic acid) was synthesized (Supplementary Figure S2A and C). The shorter linker was suitable for monolayer assembly. For C-DNA, the longer linker, 5′-NH_2_-(CH_2_)_6_-TTTT, was used. For Dex, NH_2_-PEG-NH_2_ (MW = 600) was used as the linker. The primary hydroxyl group of Dex was in advance conjugated with amino groups of polyethylene glycol (PEG; Supplementary Figure S2D) (17). Among these linkers, PEG was the longest linker, which would help to maintain the recognition function of Dex. Finally, by using the Michael addition reaction between the amine of linkers and the catechol groups of PDA (30), the functional units were modified into UCNPs@PDA in sequence (UCNPs@Ce@C-DNA&Dex, thus named UCeCD). Fourier-transform infrared spectroscopy spectra were used to testify the corresponding modification processes (Supplementary Figure S1D). The loading amount of C-DNA and PEG–Dex in UCeCD was 11.71%, suggested by the thermogravimetric analysis (Supplementary Figure S3A). Thereinto, UV–vis spectra studies demonstrated that 6.5 μM DNA was modified onto 1 mg/ml of UCNPs (Supplementary Figure S3B). Besides, the XPS analysis also confirmed that Ce, P and N all existed on the surface of UCNPs (Figure 2F and H). What’s more, the cerium ions were mainly in the +4 oxidation state, which is the key to nuclease-like activity (31). On the basis of the inductively coupled plasma mass spectrometry results, the amount of Ce was ∼6.67% anchored on the nanoparticles (Supplementary Table S1). Finally, the TEM images and hydrodynamic diameter measurements showed that UCeCD was uniform and small enough (<60 nm) to enter the nucleus (Supplementary Figure S3C–E).

### The telomeric G-overhang-targeted ability of UCeCD

**Figure 3.**
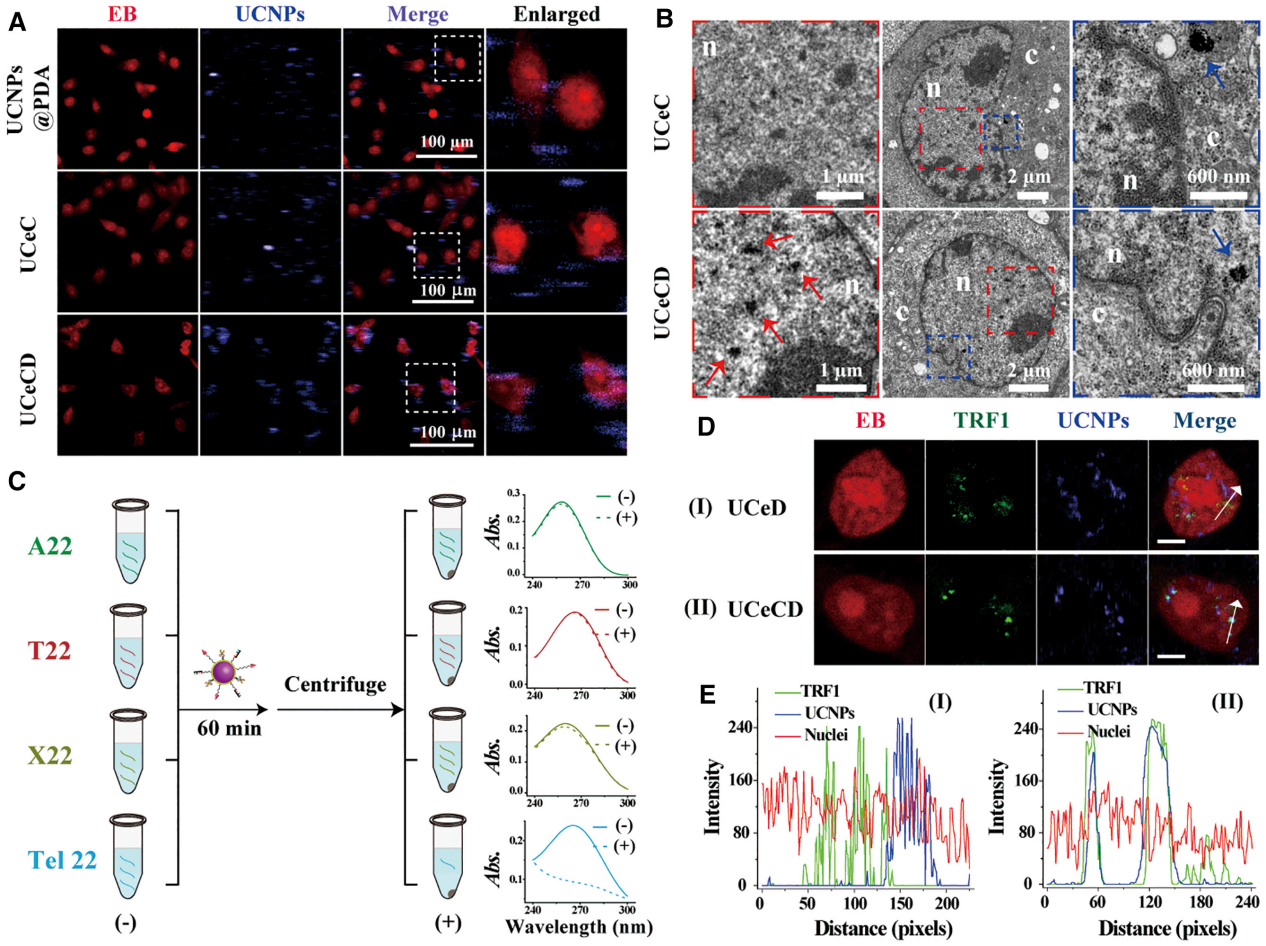
The telomeric G-overhang-targeted ability of UCeCD. (**A**) The images of MCF-7 cells after incubation with different nanoparticle solutions for 4 h. (**B**) TEM micrographs of MCF-7 cells incubated with UCeC and UCeCD. The left and right columns are higher magnification of the boxed region in the middle column (arrows: nanoparticles; n: nucleus; c: cytoplasm). (**C**) UCeCD was, respectively, mixed with four ssDNA for 60 min and centrifuged. UV–vis spectra of the respective supernatants were measured. [DNA] = 1 μM. Representative LSCM images (**D**) and line scanning profiles (**E**) of fluorescence intensity of MCF-7 cells after incubation with UCeD and UCeCD solutions for 4 h. The red fluorescence from EB was used to stain the nuclei. The blue fluorescence was from UCNPs under excitation of 980 nm laser. [UCNPs] = 50 μg/ml. Scale bars = 10 μm.

The telomeric G-overhang-targeted ability of UCeCD was validated through three steps, including nucleus targeting, telomeric DNA hybridizing and G-overhang targeting. Nuclear localization of nanoparticles was observed by laser scanning confocal microscopy (LSCM) and TEM micrographs (Figure 3A and B). The nuclei were stained red by ethidium bromide (EB), while nanoparticles were tracked by UCL signals of UCNPs excited by 980 nm laser. In contrast, the nanoparticles without Dex (UCNPs@Ce@C-DNA, named UCeC) or C-DNA (UCNPs@Ce@Dex, named UCeD) were prepared. Four hours after delivery, UCeCD was mainly localized in the nucleus. As controls, neither UCNPs@PDA nor UCeC showed obvious nuclear aggregation, revealing that Dex played a key role in nuclear targeting.

Telomeric DNA targeting of UCeCD was demonstrated by measuring DNA UV–vis spectral changes of the supernatants after centrifugation. Compared with the control nanomaterials without C-DNA, both UCeC and UCeCD exhibited higher capture efficiency (47.4 and 41.4%) in capturing telomeric DNA (Tel_22_) (Figure 3C and Supplementary Figure S4), showing that modification of C-DNA endowed UCNPs the ability to target telomeric DNA. In addition, when UCeCD was incubated with other DNA sequences, such as A_22_, T_22_ and random sequences of 22 nucleotides (X_22_) at room temperature, after centrifugation of the sample, no obvious absorbance changes of the supernatants were observed (Figure 3C), demonstrating that UCeCD could specifically target telomeric DNA.

Further, an immunofluorescence staining assay was performed in MCF-7 cells to verify the targeting ability of UCeCD for telomeric G-overhang (Figure 3D and E). TRF1 is a specialized protein bound to the telomere terminals. Confocal microscopy images and line scanning profiles of fluorescence intensity showed that, in the case of UCeCD, the green signals (from TRF1) and the blue signals (from UCNPs) were well matched with each other, displaying that most of UCeCD was co-localized with TRF1. However, UCeD tended to be randomly distributed in the nuclei, rather than co-localized with TRF1, further demonstrating that both C-DNA and Dex contributed to the G-overhang specificity of UCeCD in cell nuclei.

### The G-overhang-selective cleavage ability of UCeCD

**Figure 4.**
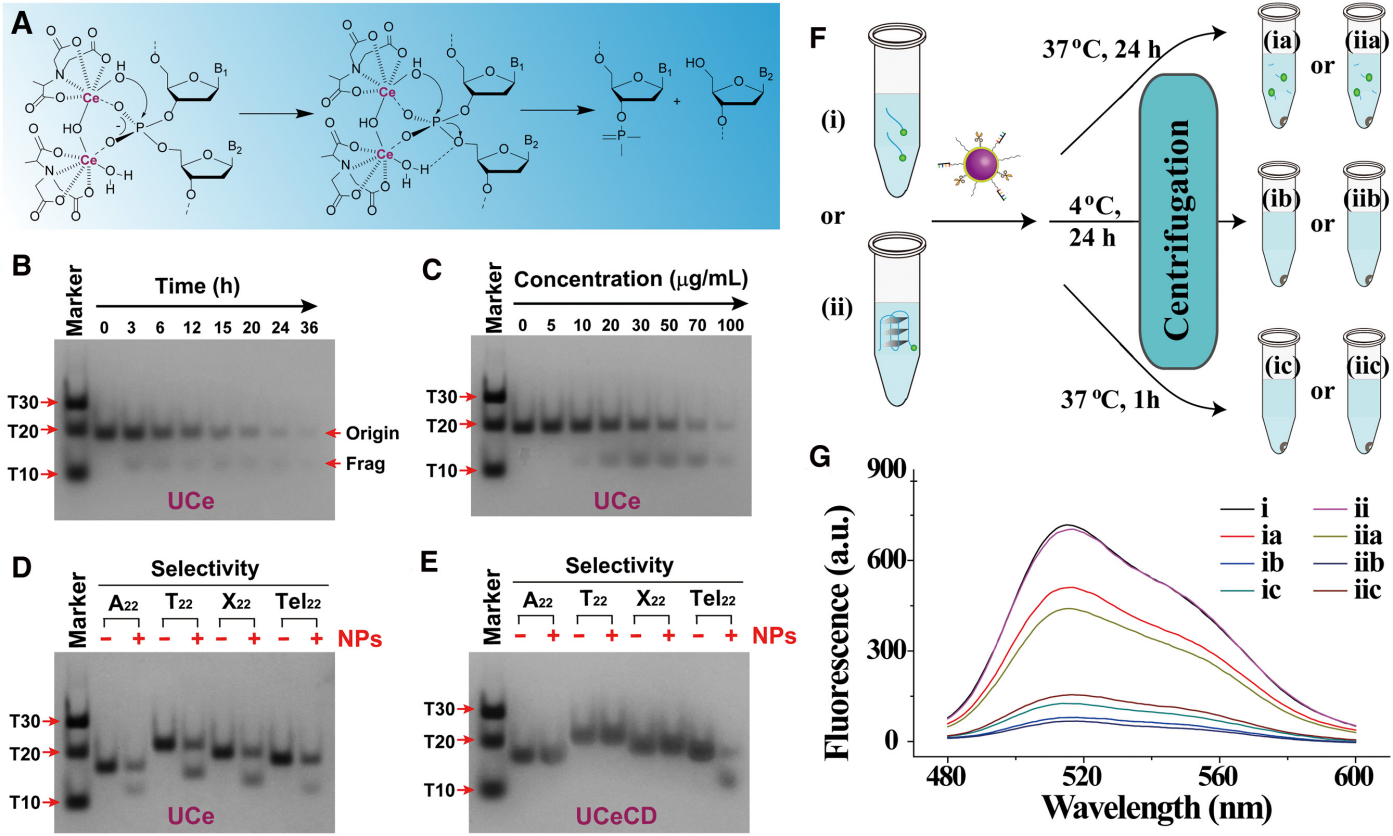
The G-overhang-selective cleavage ability of UCeCD. (**A**) Proposed mechanism of DNA cleavage by the multinuclear UCe. Denaturing gel electrophoretic analysis of DNA cleavage with different times (**B**) and various nanoparticle concentrations (**C**). [DNA] = 10 μM; [UCNPs] = 100 μg/ml. Denaturing gel electrophoretic analysis of four DNA sequences after incubation with UCe (**D**) and UCeCD (**E**) for 24 h, respectively. [UCNPs] = 120 μg/ml. Gels were run in TB buffer at room temperature. (**F**) The schematic diagram for the determination of telomeric DNA cleavage by fluorescence spectrometry. UCeCD was, respectively, mixed with FAM-Tel_16_ (i) or FAM-Tel_22_ (ii) under set conditions. The supernatants were recorded by fluorescence spectra (**G**).

To reflect the multinuclear superiority of UCNPs@Ce (named UCe), the DNA model substrate BNPP was used (Supplementary Figure S5). The *K*_m_ value of 371.4 μM calculated by the Michaelis–Menten equation showed high substrate affinity. The pseudo-first-order rate constant for BNPP cleavage was ∼1.38 × 10^−3^ s^−1^, indicating higher catalytic activity than the previously reported Ce(IV) complexes (25). Benefiting from the multivalent cooperation effects of UCe (Figure 4A), the hydrolysis of Tel_22_ DNA was analyzed by denatured gel electrophoresis experiments. Upon exposure to UCe at 37°C, Tel_22_ DNA hydrolysis was time dependent (Figure 4B). When the exposure period was increased to 24 h, most of Tel_22_ DNA was degraded into small fragments. Also, Tel_22_ DNA hydrolysis was concentration dependent (Figure 4C). In the range of 0–100 μg/ml, the higher the concentration of UCe, the better the hydrolysis effect. For UCeCD, in which both C-DNA and Dex were modified on UCe, obvious cleavage of Tel_22_ was still observed (Supplementary Figure S6), indicating UCeCD with the ability to cleave telomeric DNA. Furthermore, we verified the G-overhang-selective cleavage ability of UCeCD by employing A_22_, T_22_ and X_22_ oligonucleotides as controls. When UCe was added, all four DNA sequences were hydrolyzed (Figure 4D), indicating the poor selectivity of UCe. As comparison, for UCeCD-treated four samples, just Tel_22_ DNA displayed significant cleavage (Figure 4E), implying that UCeCD achieved selective cleavage activity toward telomeric G-overhang. Besides, FAM-labeled telomeric DNA (FAM-Tel_16_, FAM-Tel_22_) was utilized to further verify the sequence-specific cleavage ability of UCeCD (Figure 4F and G). Upon 60 min incubation, most of the fluorescence attenuation in the supernatant indicated that FAM-labeled telomeric DNA was adsorbed onto UCeCD, further supporting specific targeting telomeric DNA regardless of telomere structures (single strand or G-quadruplex). Nevertheless, after 24 h of incubation at 37°C, partial fluorescence was recovered, suggesting that telomeric DNA was fragmented and re-dissociated into the solution. Considering the self-cleavage property, we studied the stability of UCeCD. When UCeCD was stored at 4 and 37°C for a period of time, and then incubated with Tel_16_ DNA (Supplementary Figure S7), it was found that UCeCD still had a good ability to capture Tel_16_ within 12 h, even at 37°C, while the capture ability of UCeC got weakened, which indicated that the modification of PEG–Dex could effectively prevent UCeCD from self-cleavage. Therefore, UCeCD is effective within 12 h at 37°C under our experimental conditions.

### The effects of UCeCD on mammalian cancer cells

**Figure 5.**
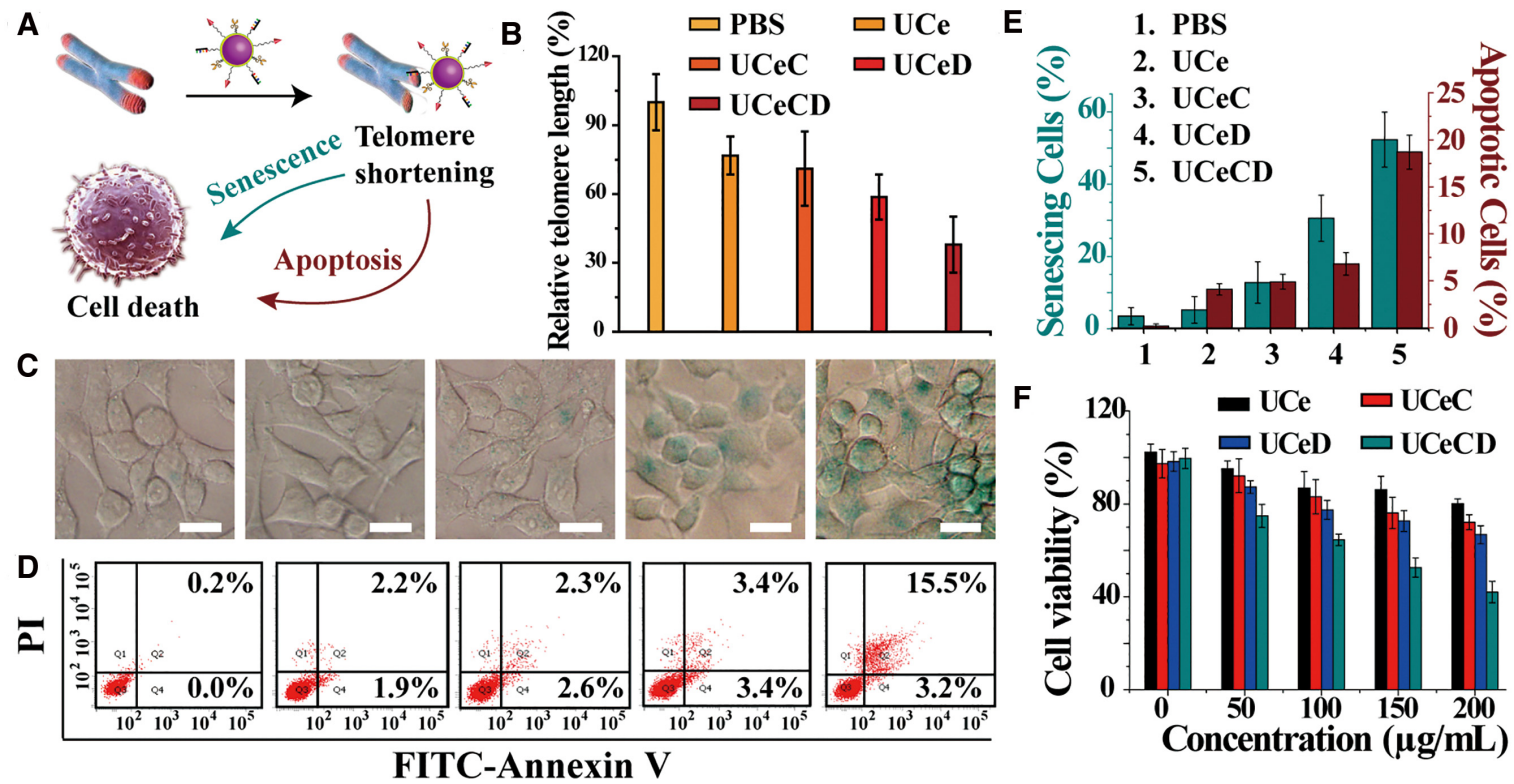
The effects of UCeCD on mammalian cancer cells. (**A**) The schematic diagram of the effects of UCeCD on MCF-7 cells. (**B**) Relative telomere length of cells measured by qRT-PCR after a 3-day treatment period. (**C**) Typical phase-contrast imaging of MCF-7 cells by a senescence-associated β-galactosidase assay. (**D**) Cell apoptosis was detected by Annexin V-FITC/PI double staining. (**E**) Quantitative analysis of the percentage of senescent and apoptotic cells. (**F**) The cell viability by MTT assay for MCF-7 cells after incubation with various concentrations of nanoparticles for 72 h. [UCNPs] = 200 μg/ml.

We studied the effects of UCeCD on mammalian cancer cells (Figure 5A). The telomere length in MCF-7 cells was measured by qRT-PCR. Relative to PBS-treated MCF-7 cells, UCeCD shortened the telomere by 62.1%, whereas UCeD alone shortened the length by 41.3% (Figure 5B). These results demonstrated that UCeCD was more effective in shortening telomere. Furthermore, DNA damage was measured by double immunofluorescence staining assays (Supplementary Figure S8). The phosphorylation of H2AX (γ-H2AX) is a common marker of DNA double-strand breaks (32). The occurrence of γ-H2AX formed foci stated that either UCeCD or UCeD induced the DNA damage response in nuclei. However, γ-H2AX formed foci were more co-localized with TRF1 formed foci after treatment with UCeCD, confirming that most of DNA damage response occurred at telomeres.

Dysfunctional telomeres, arising by critical shortening of telomeres in cells, can elicit DNA damage responses that trigger cellular senescence and apoptosis (4,11). To detect cell senescence, β-galactosidase activity, which is associated with telomere length and also a marker of cellular senescence (14), was measured after treatments. UCeCD-treated cells showed obvious β-galactosidase activity (Figure 5C). Quantitative analysis of the results revealed that UCeCD induced more than half of cell senescence (52.3%; Figure 5E). To assess cell apoptosis, flow cytometry experiments were carried out (Figure 5D). The percentage of cell apoptosis caused by UCeCD was higher than that of other groups. At last, MTT experiments were performed to observe cell viability (Figure 5F). The obvious reduced survival rate indicated that UCeCD had significant toxicity to cancer cells. At the same time, the photothermal effect accompanied by the 980 nm laser did not have a significant impact on the viability of the cells (Supplementary Figure S9).

### The anti-tumor potentials of UCeCD *in vivo*

**Figure 6.**
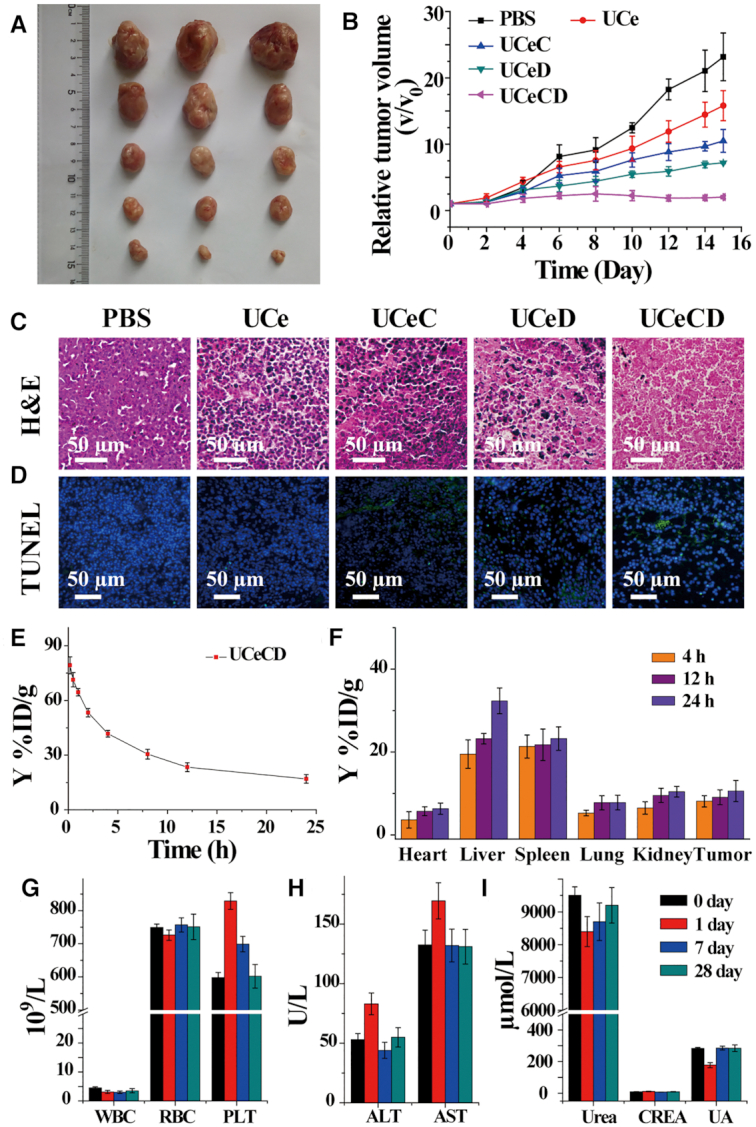
The anti-tumor potentials of UCeCD *in vivo*. (**A**) Representative photographs of the tumor dissection. (**B**) Relative tumor volume after various treatments. Micrographs of H&E stained (**C**) and TUNEL stained (**D**) tumor slices after treatments in different groups via intravenous injection. Nuclei were stained blue (DAPI staining), and apoptotic cells were stained green (TUNEL staining). The pharmacokinetic studies of UCeCD nano-hydrolase. (**E**) The blood circulation curve of intravenously injected UCeCD. (**F**) The biodistribution of UCeCD in main organs and tumors at different time points. (**G**–**I**) Blood biochemical levels and hematological parameters of the mice after treatment with UCeCD for 1, 7 and 28 days, respectively.

Inspired by the remarkable *in vitro* performance of UCeCD, we evaluated the anti-tumor potentials *in vivo*. Hepatoma 22 tumor-bearing mice were divided into five groups randomly. Different treatments were given by intravenous injection. At first, the tumor sizes were recorded, the changes of which were used to evaluate the anti-tumor effects (Figure 6A and B, and Supplementary Figure S10). The tumors of mice injected with UCeD were suppressed, which manifested the necessity of nuclear targeting. Furthermore, tumor growth was completely inhibited with the injection of UCeCD. We speculated that this could be due to the G-overhang targeting that enhanced the anti-tumor effect. Next, the treatment efficacy was further assessed by hematoxylin and eosin (H&E) staining and terminal deoxynucleotidyl transferase-mediated dUTP-biotin nick end labeling (TUNEL) staining assays (Figure 6C and D). Microscopy images of tumor sections revealed that, by the treatment of UCeCD, the number of tumor cells was greatly reduced and most damages of tumor cells were observed. Overall, all results indicated that UCeCD could inhibit tumor growth.

Finally, the potential side effects of UCeCD were investigated. MTT analysis showed that UCeCD had slightly toxic effect on normal cells under our experimental conditions (Supplementary Figure S11). The pharmacokinetics showed that the blood circulation half-life of UCeCD (*t*_1/2_ = 2.5 h, Figure 6E) was much longer than that of conventional naked nanoparticles (*t*_1/2_ = 0.33 h), which may be attributed to the PEG linker. During *in vivo* treatments, UCeCD was mainly accumulated in liver and spleen (Figure 6F). Blood biochemical levels and hematological parameters were found to be relatively normal with the control group (Figure 6G–I and Supplementary Figure S12), demonstrating that UCeCD had little toxicity to liver, spleen and kidney, and did not cause significantly systemic inflammation. Additionally, no serious weight fluctuations were presented (Supplementary Figure S13), and no obvious pathological change in major organ tissues of mice was observed (Supplementary Figure S14). Therefore, at our tested dose, UCeCD reflected biocompatibility to mice.

## CONCLUSION

In summary, we have successfully constructed a telomeric G-overhang-specific near-infrared DNA nano-hydrolase, UCeCD. It has four parts: Dex for targeting cell nuclei; C-DNA for hybridizing with G-overhang; multinuclear Ce(IV) complexes for hydrolyzing G-overhang; and UCNPs for real-time intracellular tracking. The multivalent targeted UCeCD can precisely digest telomeric G-overhang and results in telomeric DNA shortening and damage, causing cell aging and apoptosis. Animal model studies indicate that UCeCD exhibits excellent anticancer potentials *in vivo*. Our design may provide a tumor telomeric G-overhang-specific eradication strategy based on non-G-quadruplex cancer therapy, and can be easily adapted by changing the recognition modules.

## Supplementary Material

gkaa693_Supplemental_File
