# Supplementary material for: Near-infrared-traceable DNA nano-hydrolase: specific eradication of telomeric G-overhang in vivo
Source: Nucleic Acids Res. 2020 Aug 27;48(17):9986–94. doi: 10.1093/nar/gkaa693 (PMC7515709; doi:10.1093/nar/gkaa693)
Supplement: gkaa693_Supplemental_File [file gkaa693_supplemental_file.pdf]

Supplementary Materials

Near-Infrared-Traceable DNA Nano-Hydrolase: Specific Eradication of Telomeric G-Overhang *in vivo*

Yuhuan Sun, Chuanqi Zhao, Tingting Cui, Hongshuang Qin, Jingsheng Niu, Jinsong Ren and Xiaogang Qu*

* Corresponding author. Email: xqu@ciac.ac.cn

Methods
Figure S1. Material characterization of UCNPs and UCNPs@PDA.
Figure S2. 1H-NMR and mass spectrogram of NTA derivative and PEG-Dex.
Figure S3. Material characterization of UCeCD.
Figure S4. The ability of UCeCD to capture single strand DNA with UV-Vis spectra determination.
Figure S5. Nuclease-like activity of UCe based on BNPP substrate.
Figure S6. Denaturing PAGE experiments for observation of DNA cleavage.
Figure S7. The stability of UCeCD with time course.
Figure S8. Double immunofluorescence staining assays to test the DNA damage in MCF-7 cells.
Figure S9. the photothermal effect of 980 nm excitation on cell viability, senescence and apoptosis.
Figure S10. Photographs of the H22 tumor-bearing mice before or after treatments.
Figure S11-S14. The side-effect evaluation of UCeCD in vivo.
Table S1 ICP-MS and elemental analysis results of UCeCD.
Table S2. DNA sequences used in this study.

Methods

Synthesis of UCNPs (1, 2)

Briefly, $YbCl_3 \cdot 6H_2O$ (0.6 mmol), $TmCl_3 \cdot 6H_2O$ (0.01 mmol) and $YCl_3 \cdot 6H_2O$ (1.4 mmol) were added into mixture solution of octadecene (30 mL) and oleic acid (12 mL). Under nitrogen atmosphere, the mixture solution was heated to 140 ^oC. Next, as the temperature dropped to 50 ^oC, methanol solution (8 mL) including NH_4F (8.0 mmol) and NaOH (5.0 mmol) was added drop by drop. After the methanol solution was completely evaporated, the mixture was stirred at 300 ^oC for 1 h. Finally, the nanoparticles were collected by centrifugation and washed with ethanol for three times.

Synthesis of PEG-Dex (3)

Dex (68.8 mg, 0.17 mmol) and DSC (61.2 mg, 0.23 mmol) were dissolved in 5.0 mL of DMSO in the presence of TEA (65 µL, 0.46 mmol) and stirred at room temperature for 6 h. Then, this mixture was added dropwise to amino-PEG-amino (0.21 g, 0.34 mmol). After being stirred for 24 h, DMSO was removed in vacuum. Then precipitates were obtained by crystallization in isopropanol solution, and washed with cold ether. The products were dried at room temperature in vacuum to obtain amino-PEG-Dex (denoted as PEG-Dex).

Synthesis of (1S)-N-(5-amino-1-carboxypentyl)iminodiacetic acid (NTA derivative) (4)

The synthetic route of NTA derivative was realized in two steps: (i) N^{ε}-benzyloxycarbonyl-L-lysine (8.4 g) was dissolved in NaOH solution (2 M, 45 mL), and the solution was added dropwise with stirring to a cooled solution (0 °C) of bromoacetic acid (8.34 g) in NaOH solution (2 M, 30 mL) for 2 h. The mixture was stirred overnight at 25 °C and then heating for 2 h at 70 °C, and subsequently 1 M HCl (90 mL) was added to the solution. After the mixture had been cooled, the precipitate was filtered off and dried to afford a crude white powder. This was purified by further dissolution in 1 M NaOH (100 mL) and precipitation with 1 M HCl (100 mL) to give the pure (*1S*)-*N*-(5-carbobenzyloxyamino-1-carboxypentyl)iminodiacetic acid. (ii) (*1S*)-*N*-(5-carbobenzyloxyamino-1-carboxypentyl)iminodiacetic acid (6 g) was dissolved in methanol (95 mL)/H_2O (5 mL) solution, and 10% Pd/C catalyst (0.6 g) was added. The reaction was keeping stirred in H_2 at 25 °C and 760 mmHg overnight. Afterwards, the catalyst was filtered off and rinsed with H_2O (50 mL), and the solvents were removed from the filtrate to give a white paste which crystallized in pentane. The product was re-dissolved in H_2O (20 mL), and then ethanol (15 mL) was added until the solution became cloudy; after heating to give a limpid solution, the mixture was allowed to stand at -20 °C with seeds. The white crystals were filtered off and dried to afford (1S)-N-(5-amino-1-carboxypentyl)iminodiacetic acid, the structure of which was confirmed by the 1H-NMR (D_2O) spectra in the supporting information.

Preparation of PDA-Coated UCNPs (5)

The as-prepared UCNPs (20 mg) was re-dispersed in cyclohexane (2 mL). For synthesizing PDA-coated UCNPs with nano-sized PDA shell (named as UCNPs@PDA), the mixture of triton X-100 (3.6 mL), hexanol (3.6 mL), cyclohexane (14 mL), and water (680 µL) were stirred to form a homogeneous solution. Then, cyclohexane solution of UCNPs was added and being ultrasound for 30 min. After being stirred for another 30 min, dopamine hydrochloride aqueous solution (50 µL, 25 wt %) was injected into the above reaction mixture at a rate of 1.5 µL min^{-1}. Afterward, ammonium hydroxide (75 µL, 28

wt % in water) was added into mixture. After being stirred for 6 h, the nanoparticles were precipitated by adding ethanol, collected by centrifugation (10,000 rpm for 10 min) and washed with ethanol and water. Finally, the UCNPs@PDA were re-dispersed in water and dried by vacuum evaporation.

Preparation of UCe

The as-prepared UCNPs@PDA was reacted with NTA in Tris buffer (pH 8.5) under vigorous stirring for 12 h. Then, NTA-modified UCNPs@PDA (named as UCNPs@NTA) were obtained by centrifugation (10000 rpm, three times), and washed with water. Finally, the UCNPs@NTA nanoparticles were stirred with freshly prepared $Ce(NH_4)_2(NO_3)_6$ solution (in acetonitrile) for 4 h and the supernatant solution was discarded. The final nanoparticles (UCe) were purified by rinsing with water for three times.

Preparation of UCeCD

UCe (10 mg/mL) was dispersed into tris buffer (10 mM, containing 50 mM Na^+, pH 8.5). Then PEG-Dex (1 mg) was added with stirring for 6 h. Centrifugation, the precipitation obtained is re-dissolved into tris buffer (containing 50 mM Na^+, pH 6.5). Subsequently, C-DNA-amino (5 OD) was added to the mixture. The reaction lasted four hours at room temperature, followed by 48 hours at 4 ^{o}C. Finally, centrifugation and wash with water for three times. The final product is dissolved in water and stored at 4 ^{o}C.

Immunofluorescence detection

MCF-7 cells were digested into single cells with trypsin. Then the single cell suspension (1×10^4 cells) was transferred to a coverslip pre-coated with poly-L-lysine. After fixation with 4% paraformaldehyde for 30 min at room temperature, the cells were permeabilized with 0.5% Triton X-100 for 5 min, blocked with 5% bovine serum albumin at room temperature for 30 min and probed overnight with primary antibodies against γ-H2AX, and TRF1 at 4 ^{o}C. After rinsing three times with PBS for 5 min each, cells were incubated with a fluorochrome-conjugated secondary antibody, diluted in antibody dilution buffer, for 30 min at room temperature in the dark. Labeled cells were then rinsed with PBS and analyzed under a LSCM (Nikon Eclipse Ni-E, Japan).

Quantification of telomere length by using quantitative real-time polymerase chain reaction (qRT-PCR)

Genomic DNA was extracted from each cell sample, and relative telomere length was measured using a qRT-PCR method described (6). The technique measured the factor by which the sample differed from a reference DNA sample in its ratio of telomere repeat copy number to single copy gene copy number. This ratio was proportional to the average telomere length. A revised primer set (telo-F: 5-CGGTTTGTTTGGGTTTGGGTTTGGGTTTGGGTTTGGGTT-3 and telo-R: 5-GGCTTGCCTTACCCTTACCCTTACCCTTACCCTTACCCT-3) was used for telomere amplification and acidic ribosomal phosphoprotein P0 (RPLP0) gene primers (36B4F: 5-ACTGGTCTAGGACCCGAGAAG-3; 36B4R: 5-TCAATGGTGCCTCTGGAGATT-3) used as a single-copy gene reference. Each 20 μL PCR reaction included 10 μL Syber Green PCR Master Mix (Brilliant III Ultra-Fast QPCR) 100 ng genomic DNA, and primers at final concentrations: 200 nM for each telomere primer or 300 nM for each of the 36B4 primers. The amplification was performed in an Agilent MX3000P Light cycler system using the following conditions: 95 °C for 1 min, followed by 40 cycles of 95 °C for 15 s, 58 °C for 15 s, and 72 °C for 40 s. The MxPro software was used for calculation of the Ct values and the relative telomere length. The relative telomere/single copy gene

ratio (T/S value) was calculated using the formula T/S ≈ $2^{\Delta Ct}$, where ΔCt = Ct (36B4) – Ct (Tel).

Senescence-Associated β-Galactosidase Assay

MCF-7 Cells treated with nanoparticles (72 h) were washed twice in PBS, fixed in 4% formaldehyde for 30 min at room temperature, washed again in PBS, and stained by use of an X-gal solution (1 mg/mL, pH 6) for 16 h at 37°C as described in the reported method (7). Cells were viewed with an OLYMPUS BX-51 light microscope and photographed. More than 5 different fields of glass slip were randomly chosen, and over 1000 cells in the chosen fields were counted to calculate the percentage of senescent cells.

Annexin V/propidium iodide (PI) staining

The MCF-7 cells were treated with nanoparticles for 72 h. Cells were harvested and digested into single cells. After rinsing twice with PBS, 1×10^6 cells were re-suspended in 400 μL staining buffer (Annexin V-FLUOS staining Kit, BestBio, China) by mixing 5 μL of Annexin-V-FITC and incubated for 15 min at 4 °C in the dark. Then 10 μL PI was added into the mixture and continued to incubate for 5 min. Samples were analyzed immediately by flow cytometer.

TEM imaging of cellular internalization of nanoparticles

MCF-7 cells (cultured in a 6-well tissue culture plate) after 4 hours incubation with UCeCD (50 μg/mL) were fixed for 1 hour in 2.5% glutaraldehyde, post fixed in 1% osmium tetroxide. Cells were then dehydrated in a graded ethanol series and embedded in epoxy resin (Epon 812, SPI). Thin sections of the embedded cell monolayer were cut with an ultramicrotome (UC7, Leica) equipped with a diamond knife (Ultra 45° Diatome). And the sections were respectively stained with 2% uranium acetate in saturated ethanol solution and lead citrate. Images were acquired using a HITACHI HT7700 TEM.

Cell Toxicity Assays

Cell viability was measured using the (3-(4,5-dimethylthiazol-2-yl)-2,5-diphenyltetrazolium bromide, Sigma-Aldrich) MTT assays. MCF-7 cells and HEK293T cells were cultured in DMEM (Gibco BRL) medium supplemented with 5% FBS, in a 5% CO_2 humidified environment at 37 °C. For the MTT assay, cells were plated at a density of 10^4 cells per well on 96-well plates, followed by introduction of nanoparticles 24 h later. After 72 h, the cells were treated with 10 μL MTT (5 mg/mL in PBS) for 4 h at 37 °C and then were lysed in DMSO for 10 min at room temperature in the dark. Absorbance values of formazan were determined using an Ultraspec 2000 (Pharmacia Biotech) at 660 nm (reference wavelength) and 490 nm. The following equation was used to calculated cell viability: $[A]_{test}/[A]_{control} \times 100\%$, where $[A]_{test}$ was the absorbance of the test cells with treatments, and $[A]_{control}$ was the absorbance of cells treated with PBS. Three replicates were done for each treatment group.

Animal models

Healthy female Kunming mice (20-25 g) were purchased from the Laboratory Animal Center of Jilin University (Changchun, China), and handling procedures were according to the guidelines of the Regional Ethics Committee for Animal Experiments. Hepatoma 22 (H22) tumor-bearing mice were selected as the animal model to assess the antitumor effect. H22 cells were harvested from the peritonea cavity of mice 5-7 days after inoculation. Then, the cells of 2×10^5 cells were suspended in saline (about 50 μL) and subcutaneously injected into the right armpit region of mice.

For studying the therapy efficiency via intravenous injection, when the tumor volumes were about 100 mm^3, tumor-bearing mice were divided into five groups (n = 6 mice/group) randomly for different formulations: (1) saline alone; (2) UCe; (3) UCeC; (4) UCeD; and (5) UCeCD. The nanoparticles solution (500 μL, 0.5 mg mL^{-1}) was intravenously injected into mice. The tumor dimensions (length and width) and body weight were measured every other day after the treatment. The mice were sacrificed after 2 weeks post-treatment, the tumors were collected, and photos were taken.

For histology, the tumor tissues in each group were harvested from mice after the treatment. The tumor tissues were dissected to make paraffin sections for further hematoxylin and eosin (H&E) staining and terminal deoxynucleotidyl transferase-mediated dUTP-biotin nick end labeling (TUNEL) staining assays.

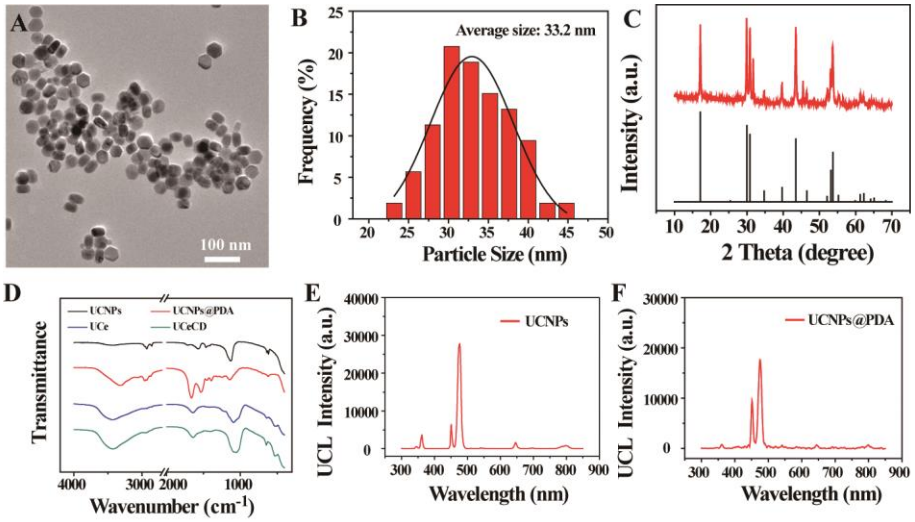

Figure S1 (A) TEM images and (B) particle size distributions of UCNPs. (C) XRD patterns of the UCNPs (red line) and the standard card (No. 28-1192) (black line). (D) FT-IR spectra analysis of nanoparticles. Absorption bands at 1464 cm^{-1} and 1567 cm^{-1} were assigned to the COOH stretching vibration of oleic acid molecules. Absorption bands at 1588 cm^{-1} (C=C aromatic ring stretching vibration), 1511 cm^{-1} (NH bending), and 3410 cm^{-1} (stretching vibration of catechol OH groups) originated from PDA. Absorption bands at 1092 cm^{-1} (C-O-C stretching) belonged to the PEG, while the glycosidic bond of C-DNA resulted to the 1400 cm^{-1} band. UCL spectra of UCNPs (E) and UCNPs@PDA (F).

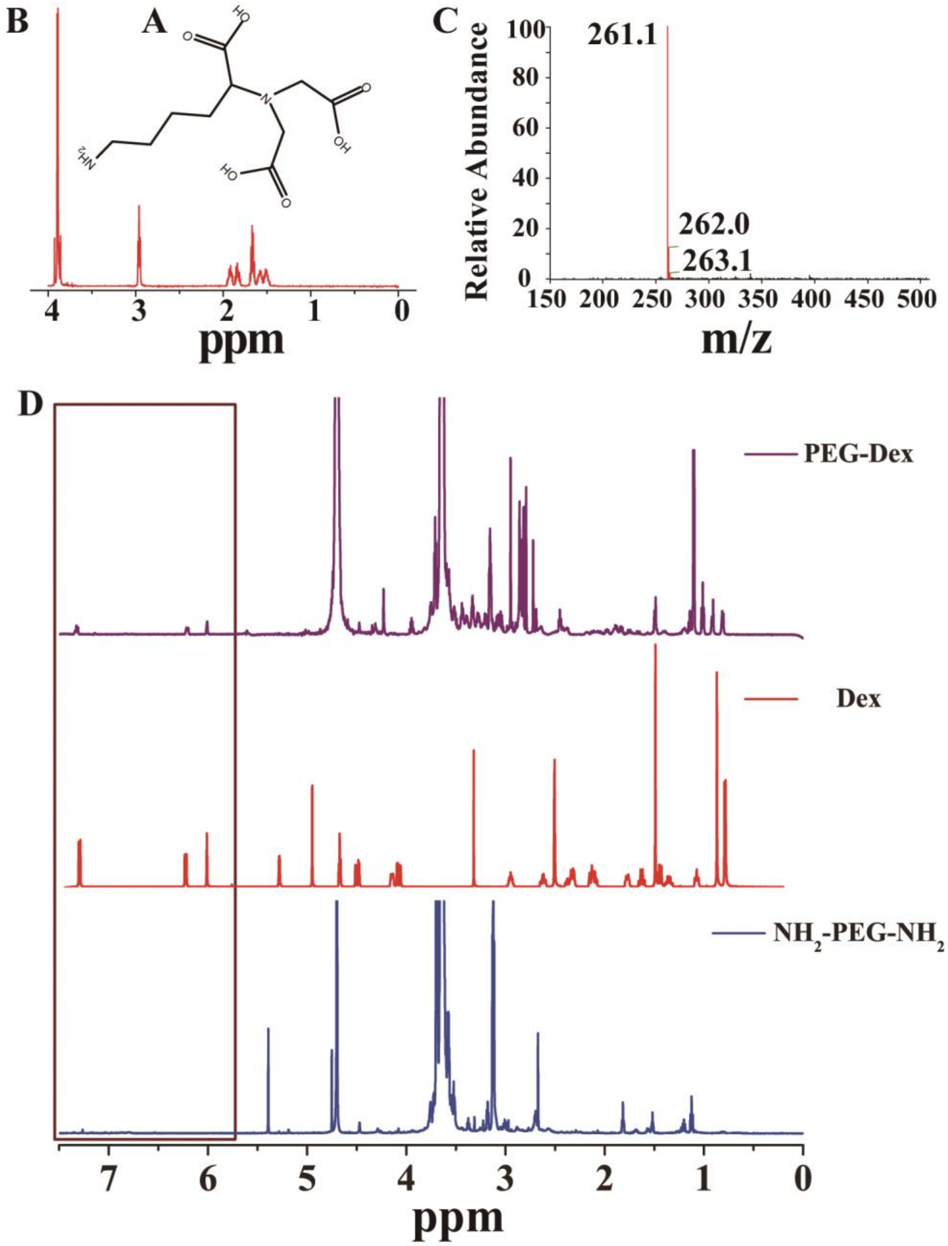

Figure S2. (A) Chemical structural formula of (1S)-N-(5-amino-1-carboxypentyl)iminodiacetic acid. (B) 1H-NMR (D_2O) and (C) mass spectrogram of the synthesized NTA derivative. δ (ppm): 3.77 (m, 5H, -COCH- and -COCH2-), 2.85 (t, 2H, -NH-CH2-CH2-), 1.76 and 1.54 (2 m, 6H, -CH2-CH2CH2CH2-CH-). (D) 1H NMR spectra of PEG-Dex, Dex, and NH_2-PEG-NH_2.

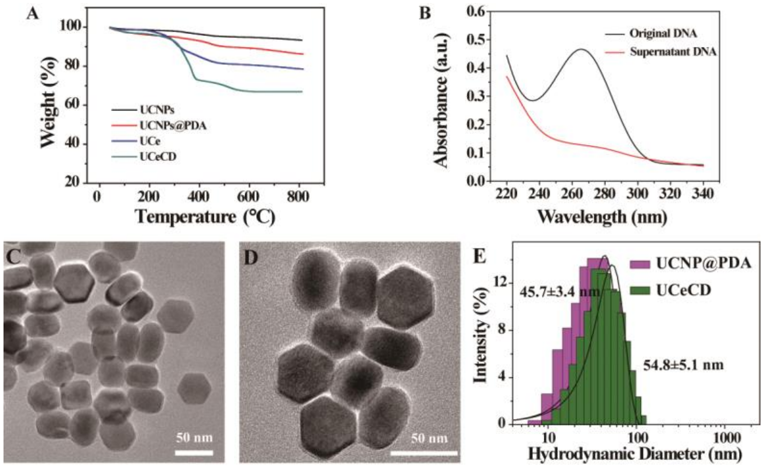

Figure S3. (A) TGA of UCNPs and modified UCNPs at a heating rate of 10 °C/min from 50 °C to 800 °C under air atmosphere. (B) UV-vis spectra of DNA solution in supernatant before and after modification. (C) and (D) TEM images of UCeCD. (E) The hydrodynamic diameter of UCNPs@PDA and UCeCD measured in water.

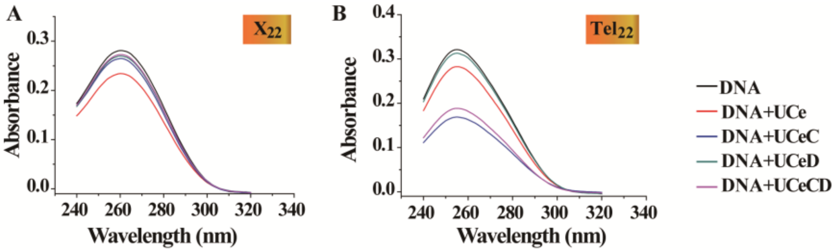

Figure S4. DNA (X_{22} or Tel_{22}) was respectively mixed with various nanoparticles for 60 minutes, and then centrifuged. Respective supernatants were taken for UV-Vis spectra determination. [DNA] = 1.3 μM.

The DNA-capture efficiency (DE) of nanomaterials was calculated as following equation:

$$DE = \frac{A_{\text{before}} - A_{\text{after}}}{A_{\text{before}}} \times 100\%$$

A_{before} means the UV absorbance value at 260 nm of origin DNA solution. A_{after} equals the UV absorbance value at 260 nm of supernatant of the blend of DNA and nanoparticles after centrifugation.

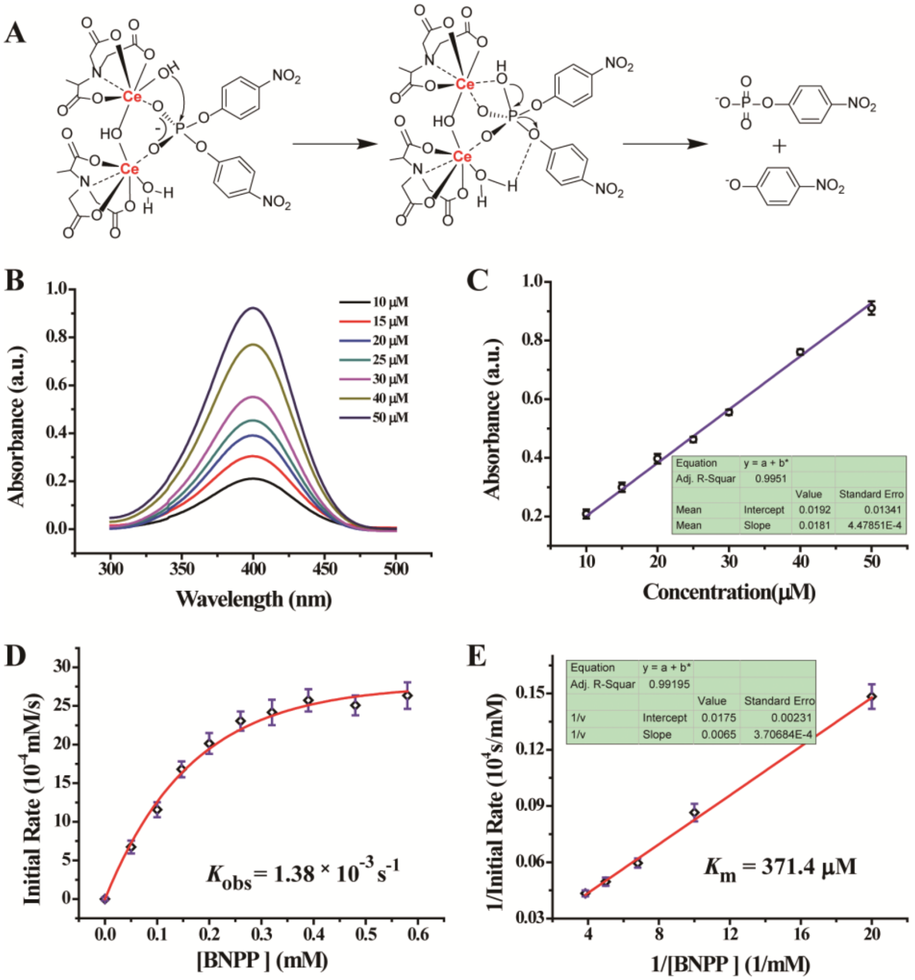

Figure S5. (A) Proposed mechanism of BNPP cleavage by the multinuclear UCe. (B) Absorption spectra of the nitrophenolate with different concentrations of 10-50 nM. (C) Standard curve of the absorbance at 400 nm as a function of nitrophenolate concentration. (D) Initial rates of BNPP cleavage by UCe as a function of the concentration of BNPP. (E) Lineweaver-Burk plot of the reciprocal of initial reaction rate versus reciprocal of the BNPP concentration. Reaction conditions: [UCNPs] = 100 μg/mL, Tris buffer (50 mM, pH = 8.0), 25 °C.

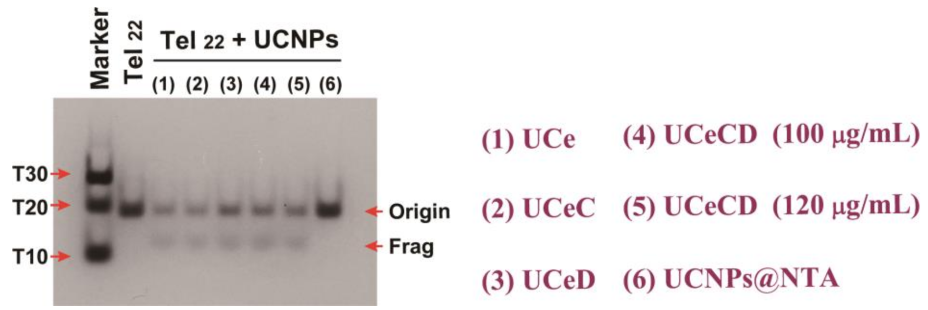

Figure S6. Denaturing PAGE for Tel_{22} after hydrolysis with modified UCNPs for 24 h. [DNA] = 10 μM. [UCNPs] = 100 μg/mL (except for lane 5). Gels were run in TB buffer at room temperature.

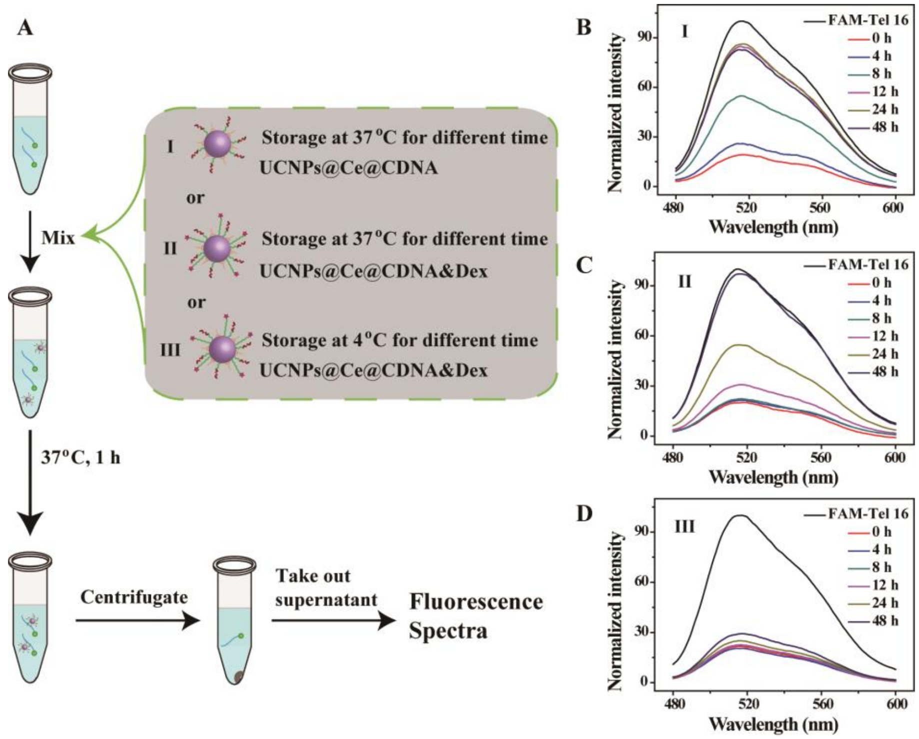

Figure S7. (A) The schematic diagram for observing the stability of UCeCD. UCeC or UCeCD was seperately stored at 37 °C or 4 °C for different times (0-48 h), and then blended with FAM-Tel_{16} for 1 hour for full binding. Thereafter, the supernatant was centrifuged, and the fluorescence spectra of the supernatant were measured as (B)-(D). From the data point of view, after 8 hours of storage at 37 °C, the ability of UCeC to bind Tel_{16} significantly decreased. UCeCD still has a strong enrichment ability after 12 hours of storage. Under 4 °C, UCeCD can be stored stably for at least 48 hours.

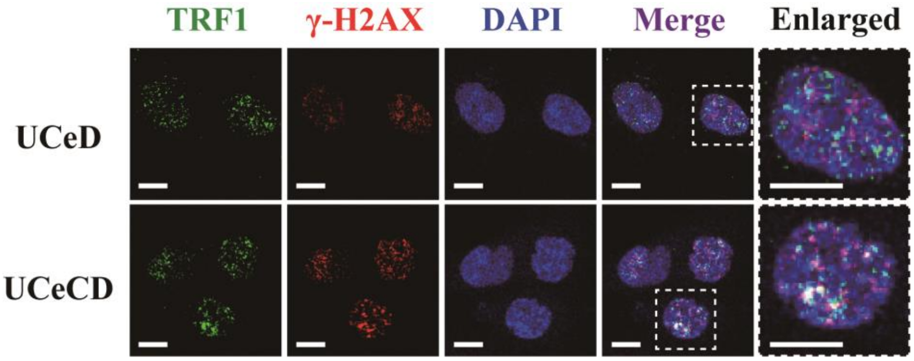

Figure S8. Double immunofluorescence staining assays to test the DNA damage in MCF-7 cells. Representative confocal images of merged TRF1 (green)/γ-H2AX (red)/DAPI (blue). MCF-7 cells were respectively treated with UCeD and UCeCD. Scale bar = 10 μm.

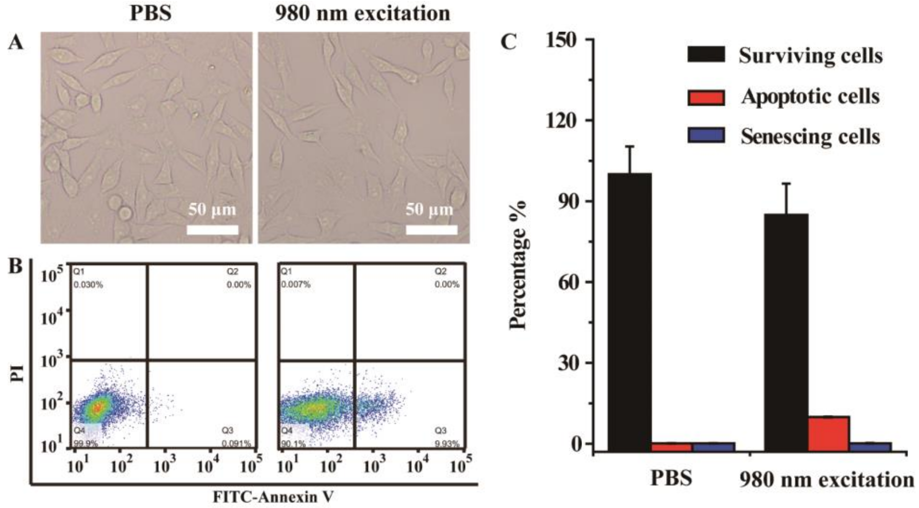

Figure S9 (A) Typical phase-contrast imaging of MCF-7 cells by a senescence-associated β-galactosidase assay. (B) Cell apoptosis was detected by Annexin V-FITC/PI double staining. (C) Quantitative analysis of the percentage of surviving cells on the basis of MTT assays, the percentage of apoptotic cells and senescing cells.

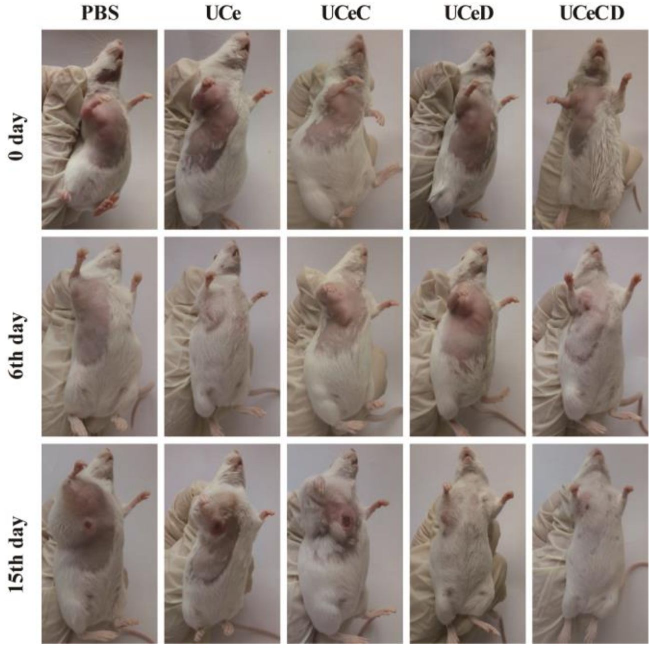

Figure S10. (A) Photographs of the H22 tumor-bearing mice before treatment, on sixth day and fifth day after the various treatments.

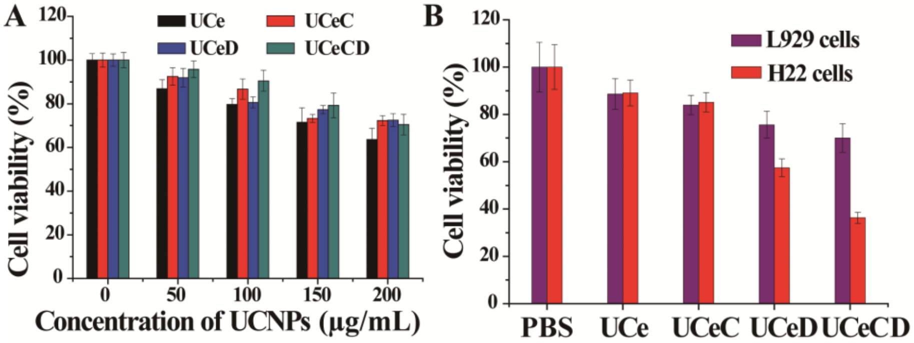

Figure S11. (A) Estimation of HEK 293T cell viability by MTT assay upon different treatments with various concentrations of nanoparticles. (B) In vitro viability of L929 cells and H22 cells in various treatments. The concentration of nanoparticles was 200 μg/mL.

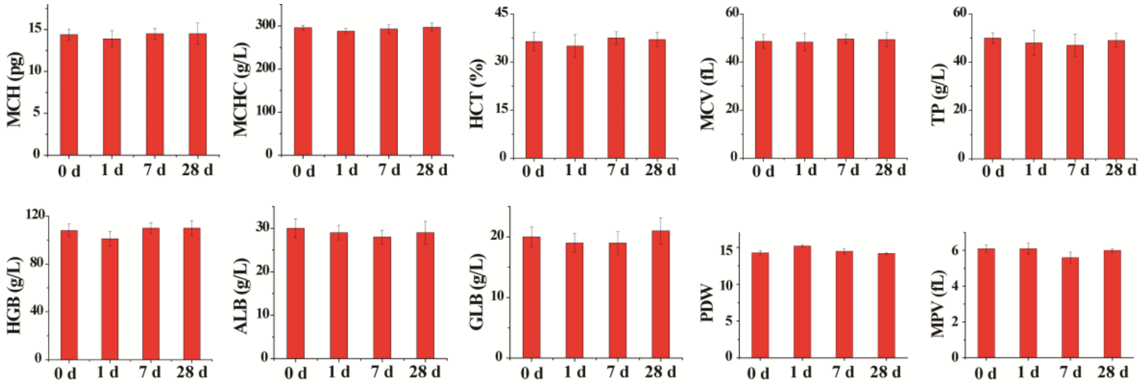

Figure S12. Blood biochemical levels and hematological parameters of the mice after treatment with UCeCD for 1, 7, and 28 days.

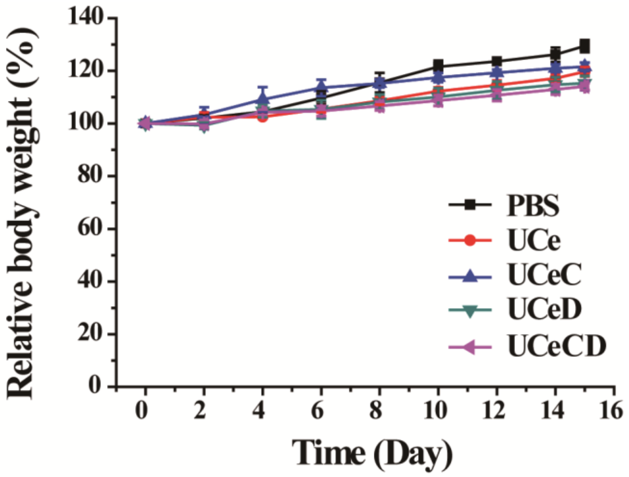

Figure S13. Body weight growth curves of tumor-bearing mice after various treatments with intravenous injection. No significant weight drop was found with all the treatment groups.

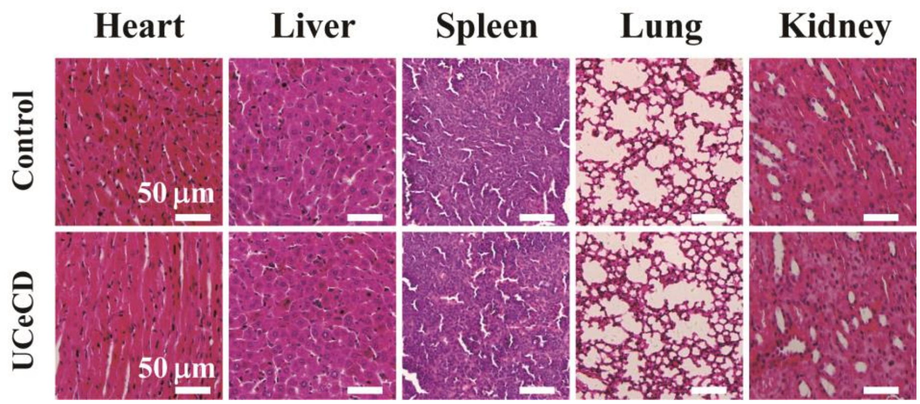

Figure S14. Histologic evaluation of tissues from mice treated with PBS (pH = 7.4) and UCeCD solution.

Table S1 ICP-MS and elemental analysis results of UCeCD.

	UCeCD
Y/%	33.1
Na/%	6.33
Yb/%	17.8
Tm/%	0.926
Ce/%	6.67
P/%	0.914

Table S2. DNA sequences used in this study.

DNA	Sequence
A_{22}	5'- AAAAAAAAAAAAAAAAAAAAAA-3'
T_{22}	5'- TTTTTTTTTTTTTTTTTTTTTT-3'
X_{22}	5'- AGTCACAGTAGTCACTAGTCAT-3'
Tel_{22}	5'- AGGGTTAGGGTTAGGGTTAGGG-3'
Tel_{16}	5'- AGGGTTAGGGTTAGGG-3'
CDNA	5'-NH_2-C_6-TTTTCCCTAACCCTAACCCTAAC CCTAACCC-3'
FAM-Tel_{22}	5'-AGGGTTAGGGTTAGGGTTAGGG-FAM-3'
FAM-Tel_{16}	5'-AGGGTTAGGGTTAGGG-FAM-3'

1. Ma, M., Gao, N., Sun, Y., Ren, J., and Qu, X. (2017) A Near-Infrared Responsive Drug Sequential Release System for Better Eradicating Amyloid Aggregates. Small, 13, 1701817.
2. Qian, H. S., Guo, H. C., Ho, P. C.-L., Mahendran, R., and Zhang, Y. (2009) Mesoporous-Silica-Coated Up-conversion Fluorescent Nanoparticles for Photodynamic Therapy. Small, 5, 2285-2290.
3. Wang, H., Li, Y., Bai, H., Shen, J., Chen, X., Ping, Y., and Tang, G. (2017) A Cooperative Dimensional Strategy for Enhanced Nucleus-Targeted Delivery of Anticancer Drugs. Adv. Funct. Mater., 27, 1700339.
4. Hochuli, E., Döbeli, H., and Schacher, A. (1987) New Metal Chelate Adsorbent Selective for Proteins and Peptides Containing Neighbouring Histidine Residues. J. Chromatogr. A, 411, 177-184.
5. Liu, F., He, X., Lei, Z., Liu, L., Zhang, J., You, H., Zhang, H., and Wang, Z. (2015) Facile Preparation of Doxorubicin-Loaded Upconversion@Polydopamine Nanoplatforms for Simultaneous in Vivo Multimodality Imaging and Chemophotothermal Synergistic Therapy. Adv. Health. Mater., 4, 559-568.
6. O'Callaghan, N. J.,Fenech, M. (2011) A Quantitative PCR Method for Measuring Absolute Telomere Length. Biol. Proced. Online, 13, 3.
7. Riou, J. F.,Guittat, L.,Mailliet, P.,Laoui, A.,Renou, E.,Petitgenet, O.,Mégnin-Chanet, F.,Hélène, C., and Mergny, J. L., (2002) Cell Senescence and Telomere Shortening Induced by a New Series of Specific G-Quadruplex DNA Ligands. Proc. Natl. Acad. Sci. USA, 99, 2672.
